# Telescope: an interactive tool for managing large-scale analysis from mobile devices

**DOI:** 10.1093/gigascience/giz163

**Published:** 2020-01-23

**Authors:** Jaqueline J Brito, Thiago Mosqueiro, Jeremy Rotman, Victor Xue, Douglas J Chapski, Juan De la Hoz, Paulo Matias, Lana S Martin, Alex Zelikovsky, Matteo Pellegrini, Serghei Mangul

**Affiliations:** Department of Clinical Pharmacy, School of Pharmacy, University of Southern California, 1985 Zonal Avenue, Los Angeles, CA 90089-9121, USA; Institute for Quantitative and Computational Biosciences, University of California Los Angeles, 611 Charles E. Young Drive East, Los Angeles, CA 90095, USA; Department of Computer Science, University of California, Los Angeles, 404 Westwood Plaza, Los Angeles, CA 90095, USA; Department of Computer Science, University of California, Los Angeles, 404 Westwood Plaza, Los Angeles, CA 90095, USA; Department of Anesthesiology, David Geffen School of Medicine at UCLA, 650 Charles E. Young Drive, Los Angeles, CA 90095, USA; Center for Neurobehavioral Genetics, University of California Los Angeles, 695 Charles E Young Dr S, Los Angeles, CA 90095, USA; Department of Computer Science, Federal University of São Carlos, km 325 Rod. Washington Luis, São Carlos, SP 13565–905, Brazil; Department of Clinical Pharmacy, School of Pharmacy, University of Southern California, 1985 Zonal Avenue, Los Angeles, CA 90089-9121, USA; Department of Computer Science, Georgia State University, 1 Park Place, Atlanta, GA 30303, USA; The Laboratory of Bioinformatics, I.M. Sechenov First Moscow State Medical University, Moscow 119991, Russia; Institute for Quantitative and Computational Biosciences, University of California Los Angeles, 611 Charles E. Young Drive East, Los Angeles, CA 90095, USA; Department of Clinical Pharmacy, School of Pharmacy, University of Southern California, 1985 Zonal Avenue, Los Angeles, CA 90089-9121, USA

**Keywords:** bioinformatics, job scheduler, high-throughput computing, bioinformatics analysis

## Abstract

**Background:**

In today's world of big data, computational analysis has become a key driver of biomedical research. High-performance computational facilities are capable of processing considerable volumes of data, yet often lack an easy-to-use interface to guide the user in supervising and adjusting bioinformatics analysis via a tablet or smartphone.

**Results:**

To address this gap we proposed Telescope, a novel tool that interfaces with high-performance computational clusters to deliver an intuitive user interface for controlling and monitoring bioinformatics analyses in real-time. By leveraging last generation technology now ubiquitous to most researchers (such as smartphones), Telescope delivers a friendly user experience and manages conectivity and encryption under the hood.

**Conclusions:**

Telescope helps to mitigate the digital divide between wet and computational laboratories in contemporary biology. By delivering convenience and ease of use through a user experience not relying on expertise with computational clusters, Telescope can help researchers close the feedback loop between bioinformatics and experimental work with minimal impact on the performance of computational tools. Telescope is freely available at https://github.com/Mangul-Lab-USC/telescope.

## Introduction

Exponential growth in the volume of available omics data has reshaped the landscape of contemporary biology, creating demand for a continuous feedback loop that seamlessly integrates experimental biology and bioinformatics [1, 2]. Life science and biomedical researchers must choose from an unprecedented diversity of software tools and datasets designed for analyzing increasingly large outputs from modern genomics and sequencing technologies, which are supported by high-performance cluster infrastructures [3]. Scientific discovery in academia and industry now relies on the seamless integration of bioinformatics tools, omics datasets, and large clusters [4–8].

Many life science and biomedical researchers lacking computational training now must learn how to use computational tools in order to process data from their experiments or seek broad patterns in omics data. Ideally, any bioinformatics analysis tool should provide an easy-to-use interface through which the researcher can run and monitor each analysis of omics data [9]. A friendly user interface for omics tools would also enable the researcher with limited computational background to monitor and adjust their analysis without manual intervention. Lack of a user interface for management tools poses an obstacle to novice users who wish to perform analysis on high-performance computing clusters [10]. The procedure of connecting to the cluster often involves a multi-step process and requires generating SSH keys or other forms of authentication. The necessity of using the UNIX command line for each step may discourage potential users.

Yet most bioinformatics tools require the researcher to spend a large amount of time manually adjusting and supervising actively running analytical tasks (referred to as jobs) via the command line in a computational pipeline. Today's high-performance computational facilities are capable of processing considerable volumes of data, but a new bottleneck has developed: their user interfaces require the researcher fluent knowledge of the command line in order to manipulate analysis in real time.

There is a pressing need to seamlessly integrate bioinformatics analysis into the experimental analysis performed by biomedical researchers, in order to expand research opportunities to individuals who lack a computational background and to reduce the time burden of any researcher who uses a computational pipeline. One example in this direction is the Galaxy Project, which provides a friendly and interactive interface to deploy simple bioinformatics pipelines [11]. Despite many advantages, Galaxy Project lacks a flexible interface to manage the analytical tasks, and many parameters related to allocating the computational resources are predefined (i.e., the number of processes is hard coded) [12].

Bridging the gap between bioinformatics and biological experimentation requires an on-the-fly job management application that is fronted by a user-friendly interface [13]. We developed Telescope to addresses this challenge. Telescope is capable of leveraging common and familiar technologies that do provide a user-friendly interface to manage jobs from any mobile device without compromising flexibility for advanced users. For example, Telescope allows users to track with their smartphones any bioinformatics tools (e.g., GATK [14]) or jobs submitted by specific platforms (e.g., Galaxy Project [14, 15]), displaying in real time the partial outputs, warnings, and error messages associated with each job. Telescope includes the following functionality:

- tracking the progress and performance of actively running bioinformatics tools;
- displaying in real time the current output of an active job;
- interacting with the computational cluster with minimal effort, allowing cancellation and/or rescheduling of jobs with different parameters, or new job queuing;
- using statistics archived from previous jobs to estimate the resources necessary for future jobs.

Telescope is designed to natively operate with a simple and straightforward interface based on Web 2.0 technology that is compatible with most modern devices (e.g., tablets and smartphones). Moreover, Telescope assumes little from the server side, requiring only the existence of a scheduling system (e.g., Sun Grid Engine [SGE], SLURM [16]) and SSH connection, both elements featured in virtually all cluster systems dedicated to high-performance computing. Because no further assumptions are made, Telescope is tuned to interfere as minimally as possible with cluster performance. We successfully tested Telescope at the University of California, Los Angeles (UCLA) campus-wide computational cluster [17], and we designed the tool for smooth integration with other cluster systems. To integrate Telescope in high-performance clusters, the technical team managing the cluster must only review Telescope's requirements.

## Related Work

Several tools exist that provide management and monitoring of bioinformatics analysis tasks, but they offer limited functionality and deployment when compared to Telescope. PHPQstat [18] and GE Web Application [19] are open-source PHP applications that provide web interfaces that allow users to monitor the status of jobs managed by SGE, a commonly used high-throughput cluster system. PHPQstat and GE Web Application are limited to use with SGE and display only details of the jobs currently running on the cluster. (Telescope includes in the display for each job additional functionalities, such as job submission and tracking history.) Virtual Desktop (VDI) [20] provides users a web-based user interface to interact with the Faculty of Arts and Sciences, Research Computing (FASRC) Cluster at Harvard University. Among other functionalities, VDI allows users to check the status of a job, edit an existing job, and submit new jobs. However, VDI is proprietary software that is limited to deployment on the FASRC Cluster; implementation details are not publicly available.

Applications of distributed processing frameworks, such as Apache Spark [21] and Hadoop MapReduce [22], can be monitored via the framework's web-based user interfaces. These tools display detailed information about each job, including the worker nodes, statuses of job stages, and memory usage. Applications such as Apache Spark, Hadoop, and MapReduce are specifically designed for each framework and are incapable of working with commonly used scheduling systems like SGE or individual cluster systems managed by universities.

Several existing tools can be used to create and monitor jobs using a web-based interface, but they support only specific programming languages or processing pipeline formats. For example, Luigi [23] is a Python module that can be used to manage jobs via the Internet. Airflow [24] allows the creation of directed acyclic graphs (DAGs) that specify a pipeline for processing of tasks; it also provides a user interface that allows users to visualize the processing status of the jobs specified by the DAGs. Compared with these tools, Telescope is a more general tool because its main objective is to leverage the common existence of scheduling systems (e.g., SGE) on clusters. Thus, Telescope is neither designed for nor restricted to a specific programming language or processing pipeline format. Telescope was initially developed to work with SGE, but it is designed to be configurable to other scheduling systems.

Finally, several tools enable an interactive approach to building and executing bioinformatics analysis tasks but lack a function that allows the user to remotely monitor jobs. Jupyter Notebooks, an open-source web application that supports the creation and sharing of live code and data visualizations, allow users to connect to clusters and run jobs using web browsers [25, 26]. However, the Jupyter Notebooks system does not allow the user to monitor jobs from a mobile device.

## Methods

**Figure 1:**
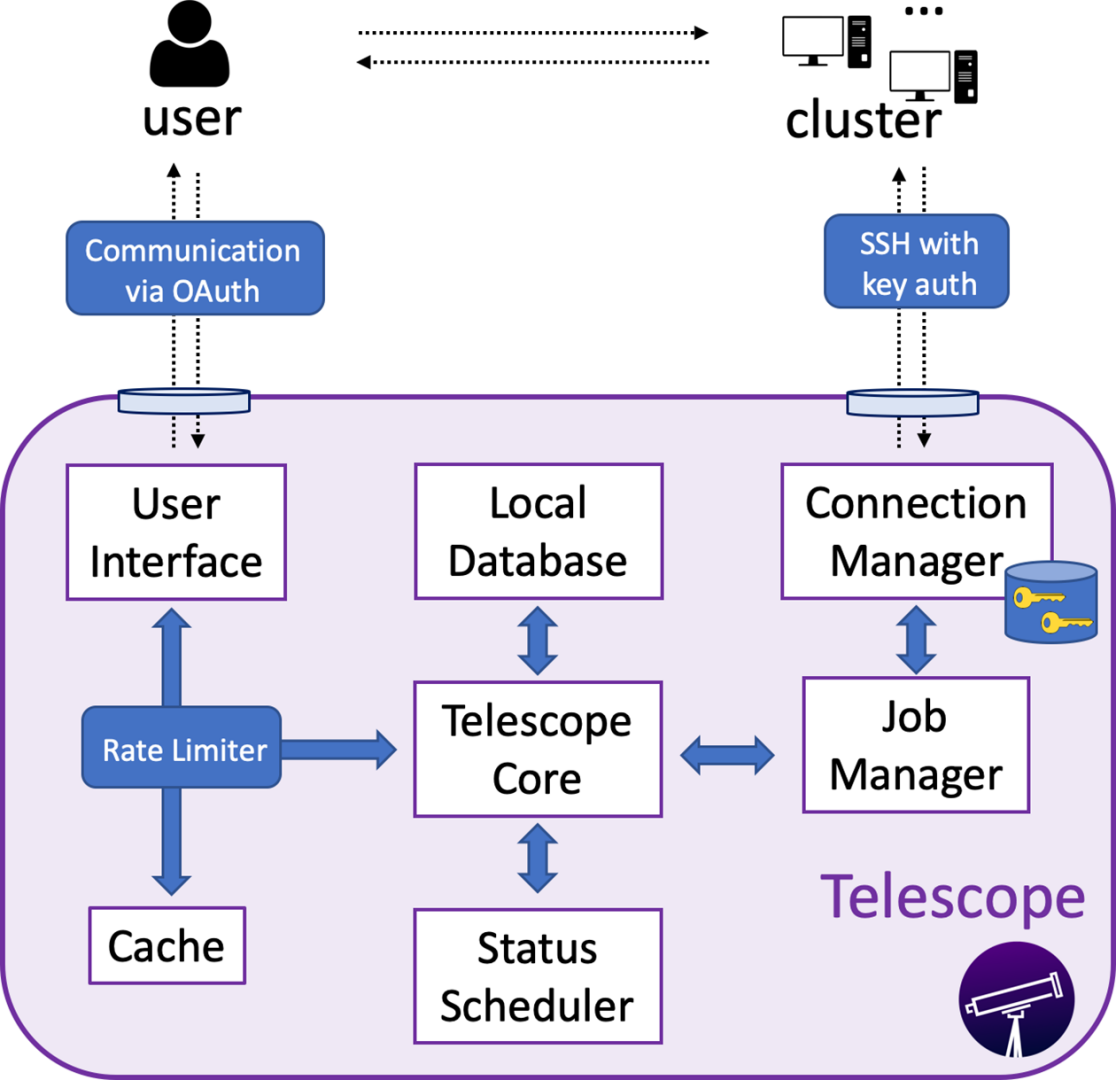
Telescope architecture. The Job Manager gathers job information by connecting to the target cluster via its Connection Manager. Telescope's Local Database keeps records of this information, which is rendered by the User Interface into a mobile-friendly web page.

Telescope comprises 2 main features (Fig. 1): a mobile-friendly user interface that relies on Web 2.0 and a connection to SSH-enabled servers. Telescope gathers job information through a Job Manager, which connects to the target cluster via the Connection Manager. Job information is then stored in Telescope's Local Database to support job analytics and a searchable history. The User Interface relies primarily on both the Local Database and the Rate Limiter to render all relevant job information into a mobile-friendly web page while limiting the impact of Telescope's interaction with the target cluster. In the following sections, we describe Telescope's key components in detail.

### Job Manager

This component handles all job requests. The Job Manager supports the operation of checking a job's status, cancelling an existing job, and creating a new job. Given a cluster's specific scheduler manager, the Job Manager leverages automated code generation based on the input data. The generated code is routed to the Connection Manager, which leverages SSH's secure code execution capability. The Telescope Core then stores the results from a completed job in the Local Database.

### Connection Manager

This component interfaces with the target cluster. The Connection Manager establishes communication via an SSH connection using key pairs for authentication. Telescope then leverages this connection to exchange discrete messages with the cluster server. As the messages are encrypted using the industry standard SSH protocol, Telescope is able to gather information without compromising the user's privacy. The Connection Manager also stores any SSH keys provided by the user.

### Local Database

The Local Database keeps records of all monitored jobs. An entry is created for each job and archives the associated job ID, job name, and user login. The Local Database also stores information regarding the requested resources (e.g., number of cores requested, memory requested), the current status of the job, and relevant metrics (e.g., elapsed time, maximum peak memory). The stored attributes can be configured for different scheduling systems (Table S1 lists the attributes in the table Job assuming a cluster with SGE). These records are retained over time to support job statistics and analytics. As these data are aggregated, the average memory and elapsed time for a given bioinformatics pipeline may be extracted as a function of the input parameters.

### Status Scheduler

For each job monitored by Telescope, the Status Scheduler periodically checks the cluster to update the Local Database with the most recent status data. The Status Scheduler is a background process and triggers update requests for all jobs in predetermined time intervals. These updates are performed in 2 steps. First, Telescope issues a query to obtain a list of all jobs running in the cluster. Then, for each active job, a new query requests detailed information. For ad hoc requests from a user, only this user's jobs are inspected.

### Telescope Core

The Telescope Core interconnects all components in the Telescope application. The User Interface and Status Scheduler generate job requests that are sent to the Telescope Core, which the Job Manager receives and handles. The results of completed job requests are propagated to update the Local Database, User Interface, and Cache. Telescope uses a Rate Limiter to restrict the rate of requests running under a specified threshold, which prevents an overload of the system running Telescope and, more importantly, the target computational cluster. (Rate limiting is a common technique used to prevent denial of service attacks [27].) Each user request must pass through this limiter before reaching the Telescope Core. When the current rate of job request exceeds the maximum threshold, additional user requests are sent to the Cache, which maintains the results of the user's last requests, rather than the Telescope Core. In addition, Telescope applies an exponential back-off algorithm that increases the time interval during which the system can accept another request from the same user.

### User Interface

**Figure 2:**
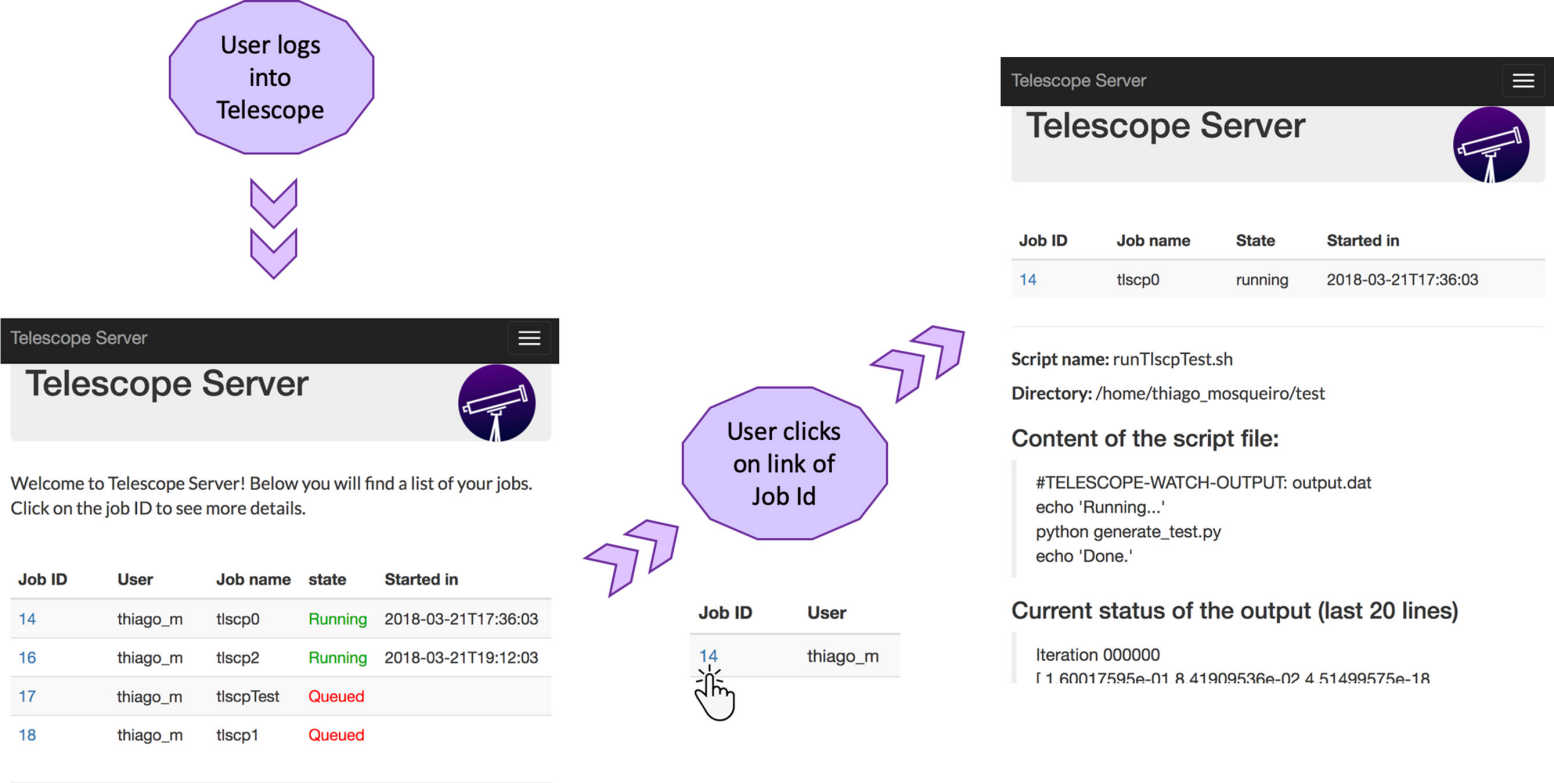
Telescope User Interface. The first screen displays the status of the jobs on the cluster. The next screen shows detailed information about the first listed job: source directory, name and content of the script file, and last lines of current task output.

Users interact with Telescope through a mobile-friendly web interface (Fig. 2). User authentication when logging into Telescope is performed via the OAuth protocol [28], which conducts verification using the user's existing accounts from popular Internet services (e.g., Google, Twitter, Facebook). After logging into Telescope, the initial web page displays a summary of all jobs actively running on the cluster under the user's account (Fig. 2, left panel), including the job identification code and name, username, current state, and starting timestamp. Each job ID is linked to a page containing more specific data for that job (Fig. 2, right panel), including the name of the script file and directory, the content of the script file, and the last few available lines from the output file. Warnings and error messages are collected from the content of logs, defined on .e files. In addition to visualizing jobs that are queued, users can also cancel or create new jobs via the User Interface. Therefore, the User Interface also supports inputting parameters to pre-defined bioinformatics pipelines.

### Security

Because Telescope handles private information and SSH keys, we designed a system that leverages industry standards for data handling and mitigation of vulnerabilities. Stored SSH private keys are encrypted using PBKDF cryptography, as recommended by the Public-Key Cryptography Standards (RFC 8018) [29]. In cases in which a private key is compromised, Telescope users may initiate a key revocation policy. Telescope currently supports SSH key revocation by deleting the compromised SSH fingerprints and updating the revocation list, a procedure that covers most Linux distributions. If a custom security policy is required by a user or cluster administration team, Telescope's modular implementation can be easily tailored by Telescope administrators.

## Discussion

Telescope interacts with the computational resources directly, at the operating system level, and spares the user from learning in-depth computer science material or devoting substantial time to manually interacting with the computational pipeline. Telescope is domain agnostic and can be used by anyone performing extensive computational analyses (e.g., deep learning, large-scale simulations for climate research).

Data retained in the database could support analytics and generate insights about job behavior, enabling users to predict resource allocation and forecast computation time. We are working on expanding the prediction feature with a simple, automated mechanism based on regular expressions that allows users to attach tags to jobs that can later be used for aggregations and analytics. For example, data from previous jobs of read alignment tools (Figs S1 and S2, Supplemental Note 1), stored in the table Job (Table S1), could have been tagged with the tool's name and the number of reads. Then, Telescope would be able to estimate the expected elapsed time and maximum amount of memory required to run these tools as a function of the number of reads. In the future, we plan to systematically collect information about the computational resources of the jobs run through Telescope. We will use recorded information to develop and provide a template that allows users to choose potential software and processing types to make better choices for resource usage.

Telescope has an intuitive user interface and demands minimal requirements from the computational cluster, making the tool appealing to users lacking a computational background, who often face a steep learning curve to operate computational resources, and to experienced users who often manage a large number of jobs and repetitive tasks. As computational clusters run Unix-based operating systems, Telescope does not completely eliminate the interaction with command line prompts but contributes to lowering the bar needed to effectively run and monitor bioinformatics analyses at scale.

We observed that Telescope users who are new users of Unix operating systems are able to, within seconds, check the status of a job and look for warning and error messages: as fast as opening their web browsers and connecting to Telescope. By addressing the challenges inherent to learning to use the command line, Telescope was designed to invite users with any level of computational experience to join the bioinformatics community.

The development of Telescope demonstrates that the current model where bioinformatics analyses are outsourced with no control during job execution (e.g., use of pre-cut pipelines wrapped in GUIs) is inefficient and prevents biomedical investigators from harnessing the true potential of their computational tools in the wet lab environment. While Telescope does not directly improve the runtime performance of bioinformatics tools, the application increases the accessibility of biomedical data analyses to the scientific community and provides for all users a tool for improving work productivity.

Real-time tracking allows biomedical researchers to access partial results—before the analytical task has been completed on a large dataset—and identify potential problems with the analysis or sequencing experiment.

The ideas and results presented in this study represent a contribution toward mitigating the digital divide in contemporary biology. By offering real-time job management tracking and control over computational clusters even on mobile devices, Telescope can help researchers accomplish a seamless feedback connection between bioinformatics and experimental work with minimal performance interference.

## Availability of Supporting Source Code and Requirements

Project name: Telescope

Project home page: https://github.com/Mangul-Lab-USC/telescope

Operating system(s): Platform independent

Programming language: Python 2.7 and 3

Other requirements: Installation of pip is required to run Telescope

License: GNU General Public License v3.0

RRID:SCR_017626

## Availability of Supporting Data and Materials

Snapshots of our code and other supporting data are available in the *GigaScience* respository, GigaDB [30].

## Additional Files

**Table S1**. Local Database's schema, explicitly listing attributes, their corresponding data types, and descriptions. These attributes correspond to the information provided by the qstat function of the scheduling system Sun Grid Engine.

**Figure S1**. Comparison of the runtime (measured by CPU time in hours) for each tool against the size of each sample (measured by the number of reads).

**Figure S2**. Comparison of the RAM (measured in gigabytes) for each tool against the size of each sample (measured by the number of reads).

giz163_GIGA-D-19-00345_Original_Submission

giz163_GIGA-D-19-00345_Revision_1

giz163_GIGA-D-19-00345_Revision_2

giz163_Response_to_Reviewer_Comments_Original_Submission

giz163_Response_to_Reviewer_Comments_Revision_1

giz163_Reviewer_1_Report_Original_Submission Riccardo Miotto -- 10/21/2019 Reviewed

giz163_Reviewer_1_Report_Revision_1 Riccardo Miotto -- 11/26/2019 Reviewed

giz163_Reviewer_2_Report_Original_Submission Shunxing Bao, Ph.D. -- 10/25/2019 Reviewed

giz163_Reviewer_2_Report_Revision_1 Shunxing Bao, Ph.D. -- 12/3/2019 Reviewed

giz163_Supplemental_File

## Abbreviations

DAG: directed acyclic graph; FASRC: Faculty of Arts and Sciences, Research Computing; GATK: Genome Analysis Toolkit; GUI: Graphical User Interface; NIH: National Institutes of Health; NSF: National Science Foundation; SGE: Sun Grid Engine; SSH: Secure Shell; UCLA: University of California, Los Angeles; VDI: Virtual Desktop.

## Competing Interests

The authors declare that they have no competing interests.

## Funding

T.M. acknowledges support from a UCLA QCBio Collaboratory Postdoctoral Fellowship and the QCBio Collaboratory community directed by Dr. Matteo Pellegrini. A.Z. has been partially supported by NSF Grants DBI-1564899 and CCF-1619110 and NIH Grant 1R01EB025022-01.

## Authors' Contributions

T.M. proposed and scoped the project. J.J.B. and T.M. developed the software presented in this article and were major contributors in writing the manuscript. J.R., V.X., D.J.C., J.D.H., P.M., L.S.M., A.Z., and M.P. contributed to portions of the code and in writing the manuscript. S.M. led the project and contributed in writing the manuscript.
