# Supplementary material for: Telescope: an interactive tool for managing large-scale analysis from mobile devices
Source: Gigascience. 2020 Jan 23;9(1):giz163. doi: 10.1093/gigascience/giz163 (PMC6977584; doi:10.1093/gigascience/giz163)
Supplement: giz163_GIGA-D-19-00345_Original_Submission [file giz163_giga-d-19-00345_original_submission.pdf]

GigaScience

Telescope: an interactive tool for managing large scale analysis from mobile devices

--Manuscript Draft--

**Manuscript Number:**	GIGA-D-19-00345
**Full Title:**	Telescope: an interactive tool for managing large scale analysis from mobile devices
**Article Type:**	Technical Note
**Funding Information:**	
**Abstract:**	In today's world of big data, computational analysis has become a key driver of biomedical research. High-performance computational facilities are capable of processing considerable volumes of data, yet often lack an easy-to-use interface to guide the user in supervising and adjusting bioinformatics analysis via a tablet or smartphone. Telescope is a novel tool that interfaces with high-performance computational clusters to deliver an intuitive user interface for controlling and monitoring bioinformatics analyses in real-time. Telescope provides a user-friendly method for integrating computational analyses with experimental biomedical research. Telescope is freely available at https://github.com/Mangul-Lab-USC/telescope .
**Corresponding Author:**	Lana S. Martin, Ph.D.<br>University of California School of Pharmacy<br>Los Angeles, CA UNITED STATES
**Corresponding Author Secondary Information:**	
**Corresponding Author's Institution:**	University of California School of Pharmacy
**Corresponding Author's Secondary Institution:**	
**First Author:**	Jaque Brito, Ph.D.
**First Author Secondary Information:**	
**Order of Authors:**	Jaque Brito, Ph.D.
	Thiago Mosqueiro, Ph.D.
	Douglas J. Chapski, Ph.D.
	Juan De la Hoz, Ph.D.
	Paulo Matias, Ph.D.
	Lana S. Martin, Ph.D.
	Matteo Pellegrini, Ph.D.
	Serghei Mangul, Ph.D.
**Order of Authors Secondary Information:**	
**Additional Information:**	
**Question**	**Response**
Are you submitting this manuscript to a special series or article collection?	No
**Experimental design and statistics**<br><br>Full details of the experimental design and statistical methods used should be given in the Methods section, as detailed in our	Yes

Powered by Editorial Manager® and ProduXion Manager® from Aries Systems Corporation

Minimum Standards Reporting Checklist. Information essential to interpreting the data presented should be made available in the figure legends.<br><br>Have you included all the information requested in your manuscript?	
**Resources**<br><br>A description of all resources used, including antibodies, cell lines, animals and software tools, with enough information to allow them to be uniquely identified, should be included in the Methods section. Authors are strongly encouraged to cite Research Resource Identifiers (RRIDs) for antibodies, model organisms and tools, where possible.<br><br>Have you included the information requested as detailed in our Minimum Standards Reporting Checklist?	Yes
**Availability of data and materials**<br><br>All datasets and code on which the conclusions of the paper rely must be either included in your submission or deposited in publicly available repositories (where available and ethically appropriate), referencing such data using a unique identifier in the references and in the "Availability of Data and Materials" section of your manuscript.<br><br>Have you have met the above requirement as detailed in our Minimum Standards Reporting Checklist?	Yes

Manuscript
Click here to download Manuscript Telescope - Technical Note - Giga Science.docx

{Technical Note}

Telescope: an interactive tool for managing large scale analysis from mobile devices

Jaqueline J. Brito[1,†*], Thiago Mosqueiro[2,†], Douglas J. Chapski[3], Juan De la Hoz[4], Paulo Matias[5], Lana S. Martin[6], Matteo Pellegrini[2], Serghei Mangul[6*]

[1]Institute of Mathematics and Computer Science, University of São Paulo, São Carlos, 400 Trabalhador São-carlense Avenue, São Carlos, SP, 13566-590, Brazil. [2]Institute for Quantitative and Computational Biosciences, University of California Los Angeles, 611 Charles E. Young Drive East, Los Angeles, CA, 90095, USA. [3]Department of Anesthesiology, David Geffen School of Medicine at UCLA, 650 Charles E. Young Drive, Los Angeles, CA, 90095, USA. [4]Center for Neurobehavioral Genetics, University of California Los Angeles, 695 Charles E Young Dr S, Los Angeles, CA, 90095, USA. [5]Department of Computer Science, Federal University of São Carlos, km 325 Rod. Washington Luis, São Carlos, SP 13565-905, Brazil. [6]Department of Clinical Pharmacy, School of Pharmacy, University of Southern California 1985 Zonal Avenue Los Angeles, CA 90089-9121

[†]These authors contributed equally to this work.

*Correspondence: jjbrito@icmc.usp.br; mangul@usc.edu

Abstract

In today's world of big data, computational analysis has become a key driver of biomedical research. High-performance computational facilities are capable of processing considerable volumes of data, yet often lack an easy-to-use interface to guide the user in supervising and adjusting bioinformatics analysis via a tablet or smartphone. Telescope is a novel tool that interfaces with high-performance computational clusters to deliver an intuitive user interface for controlling and monitoring bioinformatics analyses in real-time. Telescope provides a user-friendly method for integrating computational analyses with experimental biomedical research. Telescope is freely available at https://github.com/Mangul-Lab-USC/telescope.

Keywords

Bioinformatics, software, data analysis management

Main Text

Findings

Over the past decade, exponential growth in the volume of omics data has reshaped the landscape of contemporary biology, creating demand for a continuous feedback loop that seamlesssly integrates experimental biology and bioinformatics[1,2]. Specifically, the rapid advancement of genomics and sequencing technologies has generated an unprecedented diversity of software tools and datasets for analytical use in computational biology[3]. The combination of such tools and datasets into seamless bioinformatics pipelines, harnessing the computational power of large cluster

infrastructures, has become a cornerstone of new scientific advancements and innovation in industry and academia[4–8]. Thus, learning how to leverage computational facilities with experimental work has proven essential to laying the groundwork for life sciences and biomedical research. One example in this direction is the Galaxy Project, which provides a friendly and interactive interface to deploy simple bioinformatics pipelines[9].

While today's high-performance computational facilities can process considerable volumes of data, their user interfaces require fluent knowledge of the command line to supervise and adjust executed bioinformatics analyses in real time. The Galaxy Project is extremely popular and crucial to research in many bioinformatics labs, but the platform presents several limitations to management of analytical tasks. First, the user interface often constrains how computational resources are used (i.e., the number of processes is hard coded[10]). Second, the on-demand scheduling systems widely used in large computational facilities, such as Sun Grid Engine[11], only provide command-line interfaces, which present a steep learning curve to novice users and are not always the most efficient option, even for experienced users. The current model where bioinformatics analyses are outsourced with no control during job execution (for example, use of pre-cut pipelines wrapped in Graphical User Interfaces) is inefficient and prevents biomedical investigators from exploiting the true potential of their computational tools in the wet lab environment.

Bridging the gap between bioinformatics and biological experimentation depends on the development of on-the-fly job management seamlessly incorporated into a user-friendly interface[12]. Telescope addresses this challenge by proposing a secure and

simple tool capable of leveraging common and familiar technologies that provides a user-friendly interface to manage jobs from any device, including smartphones, without limiting advanced users. Telescope is an interactive tool that interfaces with high-performance computational clusters to deliver an intuitive interface that allows the user to control and monitor bioinformatics analyses via mobile devices. For example, Telescope allows users to track with their smartphones any bioinformatics tools (e.g., GATK[13]) or jobs submitted by specific platforms (e.g., Galaxy Project[14]), displaying in real-time partial outputs, warnings, and error messages. Telescope includes the following functionality:

- tracking of the progress and performance of running bioinformatics tools, displaying the current output in real-time in the browser;
- interaction with the computational cluster with minimal effort, allowing cancellation and/or rescheduling of jobs with different parameters, or new job queuing;
- use of archived statistics about previous jobs to estimate the resources necessary for future jobs.

Telescope was designed to natively operate with a simple and straightforward interface using Web 2.0 technology compatible with most modern devices (e.g., tablets and smartphones). Moreover, Telescope assumes very little from the server side: the existence of a scheduling system (e.g., Sun Grid Engine, SLURM[15], etc.) and SSH connection, both featured in virtually all cluster systems dedicated to high-performance computing. As no further assumptions are made, Telescope is tuned to

interfere as minimally as possible with cluster performance. We successfully tested Telescope at UCLA's campus-wide computational cluster[16].

Methods

Telescope provides two interfaces (Figure 1): a mobile-friendly user interface that relies on Web 2.0 and secure browsing protocols to allow an intuitive user experience; and a connection to SSH-enabled servers. Telescope gathers job information through a Job Manager that connects to the target cluster via the Connection Manager. Job information is then stored in Telescope's Local Database to support job analytics and searchable history. The User Interface relies primarily on both the Local Database and Rate Limiter to render all relevant job information into a (mobile-friendly) web page while limiting the impact of Telescope's interaction with the target cluster. In the following sections, we describe Telescope's key components in detail.

Job Manager. This component handles all job requests. The Job Manager supports the operations of checking a job's status, cancelation of existing jobs, and the creation of new jobs. Given a cluster's specific scheduler manager, it leverages automated code generation based on the input data. The generated code is routed to the Connection Manager, which leverages SSH's secure code execution capability. The results are then stored in the Local Database by Telescope Core.

Connection Manager. This component interfaces with the target cluster. It establishes communication via an SSH connection using key pairs for authentication. Telescope then leverages this connection to exchange discrete messages with the

cluster server. As the messages are encrypted using the industry-standard SSH protocol, Telescope is able to gather information without compromising the user's privacy. The Connection Manager also stores the SSH keys provided by the users.

Local Database. The Local Database keeps records of all monitored jobs. Detailed information about each job is represented by entries composed of several attributes, such as information that identifies the job (e.g., job id, job name, user login). In addition, the Local Database also stores information regarding the requested resources (e.g., number of cores requested, memory requested), the current status of the job, and metrics (e.g., elapsed time, max peak memory). The stored attributes can be configured for different scheduling systems (Table S1 lists the attributes in the table Job assuming a cluster with Sun Grid Engine). These records are retained over time to support job statistics and analytics. As this data is aggregated, the average memory and elapsed time for a given bioinformatics pipeline may be extracted as a function of the input parameters.

Status Scheduler. Telescope periodically checks the cluster to update the Local Database with the most recent status data from each job being monitored. The Status Scheduler is a background process and triggers update requests for all jobs in predetermined time intervals. These updates can be performed using two strategies. The first uses a common user who can query the necessary information of all users. For instance, the qstat command from Sun Grid Engine returns information about all queued jobs to any user. The second strategy issues multiple queries (one specific to each user) to the cluster, which also have access to their respective raw data. The first strategy has a low impact on the cluster as it generates a smaller number of queries.

On the other hand, the second method provides more job details because it can access all job data from each user. Telescope sets the second option as default.

Telescope Core. The core interconnects all components in the Telescope application. The User Interface and Status Scheduler generate job requests that are sent to the Telescope Core, which are then forwarded to and handled by the Job Manager. The results of such requests are propagated to update the Local Database, User Interface, and Cache. Telescope employs a Rate Limiter to keep the rate of requests running under a specified threshold, to prevent the overload of the system running Telescope and, more importantly, the target computational cluster. Rate limiting is a common technique used to prevent denial of service (DoS) attacks[18]. Therefore, user requests pass through this limiter before they reach the Telescope Core. When the current rate is above the maximum threshold, user requests are not sent to the Telescope Core. In this case, the Cache, which maintains the results of the user's last requests, responds to requests. Moreover, Telescope applies an exponential back-off algorithm that increases the time interval at which the system accepts another request from the same user.

User Interface. Users interact with Telescope through a mobile-friendly web interface (Fig. 2). The authentication into Telescope is performed via the OAuth protocol[19], supporting authentication through existing accounts from popular services (e.g., Google, Twitter, Facebook, etc.). After logging in, the initial web page displays a summary of the user's jobs running on the cluster (Fig. 2, left panel), including the job id and name, username, current state, and starting timestamp. Each job id is a link to a personalized job information page containing more specific data (Fig. 2, right

panel), including the name of the script file and directory, the content of the script file, and the last few available lines from the output file. Besides visualizing jobs that are queued, users can also cancel or create new jobs. Therefore, the User Interface supports the ingestion of parameters to run pre-defined bioinformatics pipelines.

Security. Because Telescope handles private information and SSH keys, we designed a system that leverages industry standards for data handling and compromises response. Stored SSH private keys are encrypted using PBKDF cryptography, as recommended by the Public-Key Cryptography Standards (RFC 8018)[17]. In case a private key is compromised, Telescope users may trigger a revocation process, which sets off a revocation policy. Telescope currently supports SSH key revocation by deleting SSH fingerprints and adding to key revocation list, which should cover most Linux distributions. If a custom policy is in place, Telescope's modular implementation can be easily tailored.

Discussion

Our study highlights an emergent need for modern, secure and elegant solutions to integrate the use of bioinformatics tools into the experimental environment of biomedical research. Real-time tracking allows biomedical researchers to assess partial results and identify possible problems with, for instance, the analyses of sequencing experiments. Telescope interacts with the computational resources directly, sparing the user from learning in-depth computer science material and allowing biomedical researchers to leverage the power of large computational facilities in a user-friendly manner (i.e., using their own smartphones or any personal mobile device). Telescope's simple user interface and minimal requirements from the

computational cluster make the tool appealing not only to users with minimal computational background, who often face a steep learning curve to operate computational resources, but also to experienced users who often manage a large number of jobs and repetitive tasks. Additionally, retained data in the database can be rolled up to support useful analytics about job behavior. For example, data from table Job (Table S1) can easily provide the expected elapsed time for GATK as a function of the data size by grouping by previous job sizes. Further, a simple recommendation system could leverage Telescope's stored data to provide estimates of elapsed time, number of CPUs or needed memory to run a job of a given size. Finally, Telescope is domain agnostic and can be used by anyone performing extensive computational analyses (e.g. deep learning, large-scale simulations for climate research).

The ideas and results presented in this study represent a contribution toward mitigating the digital divide in contemporary biology. By offering real-time job management tracking and control over computational clusters even on mobile devices, Telescope can help researchers to accomplish a seamless feedback connection between bioinformatics and experimental work with minimal performance interference.

Declarations

Ethics approval and consent to participate

Not applicable.

Consent for publication

Not applicable.

Availability of data and materials

The software presented in this paper is freely available at https://github.com/Mangul-Lab-USC/telescope.

Competing interests

The authors declare that they have no competing interests.

Funding

T.M. and S.M. acknowledge support from a QCB Collaboratory Postdoctoral Fellowship and the QCB Collaboratory community directed by Dr. Matteo Pellegrini.

Authors' contributions

T.M. proposed and scoped the project. J.J.B. and T.M. developed the software presented in this paper and were major contributors in writing the manuscript. D.J.C., J.D.H., P.M., L.M., and M.P. contributed to portions of the code and in writing the manuscript. S.M. lead the project and contributed in writing the manuscript.

References

[1] Markowetz F. All biology is computational biology. PLoS biology. 2017 Mar 9;15(3):e2002050.

[2] Mangul S. Interpreting and integrating big data in the life sciences. Emerging Topics in Life Sciences. 2019 Aug 16;3(4):335-41.

[3] Wren JD. Bioinformatics programs are 31-fold over-represented among the highest impact scientific papers of the past two decades. Bioinformatics. 2016 May 5;32(17):2686-91.

[4] Bulterys PL, Toesca IJ, Norris MH, Maloy JP, Fitz-Gibbon ST, France B, Toffig B, Morselli M, Somprasong N, Pellegrini M, Schweizer HP. An in situ high-throughput screen identifies inhibitors of intracellular Burkholderia pseudomallei with therapeutic efficacy. Proceedings of the National Academy of Sciences. 2019 Sep 10;116(37):18597-606.

[5] Mack JJ, Mosqueiro TS, Archer BJ, Jones WM, Sunshine H, Faas GC, Briot A, Aragón RL, Su T, Romay MC, McDonald AI. NOTCH1 is a mechanosensor in adult arteries. Nature communications. 2017 Nov 20;8(1):1620.

[6] Cook CN, Mosqueiro T, Brent CS, Ozturk C, Gadau J, Pinter- Wollman N, Smith BH. Individual differences in learning and biogenic amine levels influence the behavioural division between foraging honeybee scouts and recruits. Journal of Animal Ecology. 2019 Feb;88(2):236-46.

[7] Beal J, Haddock-Angelli T, Gershater M, De Mora K, Lizarazo M, Hollenhorst J, Rettberg R. Reproducibility of fluorescent expression from engineered biological constructs in E. coli. PLoS One. 2016 Mar 3;11(3):e0150182.

[8] Mangul S, Yang HT, Strauli N, Gruhl F, Porath HT, Hsieh K, Chen L, Daley T, Christenson S, Wesolowska-Andersen A, Spreafico R. ROP: dumpster diving in RNA-sequencing to find the source of 1 trillion reads across diverse adult human tissues. Genome biology. 2018 Dec;19(1):36.

[9] Børnich C, Grytten I, Hovig E, Paulsen J, Čech M, Sandve GK. Galaxy Portal: interacting with the galaxy platform through mobile devices. Bioinformatics. 2016 Jan 27;32(11):1743-5.

[10] The Galaxy Project Documentation. Available at: https://galaxyproject.org/community/galaxy-admins/surveys/2012/#authentication-user-and-job-management-limitations. (Accessed: 14th September 2019).
[11] Gentzsch W. Sun grid engine: Towards creating a compute power grid. InProceedings First IEEE/ACM International Symposium on Cluster Computing and the Grid 2001 May 15 (pp. 35-36). IEEE.
[12] Mangul S, Mosqueiro T, Abdill RJ, Duong D, Mitchell K, Sarwal V, Hill B, Brito J, Littman RJ, Statz B, Lam AK. Challenges and recommendations to improve the installability and archival stability of omics computational tools. PLoS biology. 2019 Jun 20;17(6):e3000333.
[13] Data Sciences Platform @ Broad Institute. GATK | Home. Available at: https://software.broadinstitute.org/gatk/. (Accessed: 12th September 2019)
[14] Galaxy Community Hub. Available at: https://galaxyproject.org/. (Accessed: 14th September 2019).
[15] Yoo AB, Jette MA, Grondona M. Slurm: Simple linux utility for resource management. InWorkshop on Job Scheduling Strategies for Parallel Processing 2003 Jun 24 (pp. 44-60). Springer, Berlin, Heidelberg.
[16] About Hoffman2 - Institute for Digital Research and Education. Available at: https://idre.ucla.edu/hoffman2. (Accessed: 14th September 2019).
[17] Moriarty K, Kaliski B, Rusch A. PKCS# 5: password-based cryptography specification version 2.1.
[18] Zargar ST, Joshi J, Tipper D. A survey of defense mechanisms against distributed denial of service (DDoS) flooding attacks. IEEE communications surveys & tutorials. 2013 Mar 28;15(4):2046-69.
[19] The OAuth 2.0 Authorization Framework. (2012). doi:10.17487/rfc6749.

{Figure Legends}

Figure 1. Telescope Architecture. The Job Manager gathers job information by connecting to the target cluster via its Connection Manager. Telescope's Local Database keeps records of this information, which is rendered by the User Interface into a (mobile-friendly) web page.

Figure 2. Telescope User Interface. The first screen displays the status of the jobs on the cluster. The next screen shows detailed information about the first listed job: source directory, name and content of the script file, and last lines of current task output.

Figures

Figure 1.

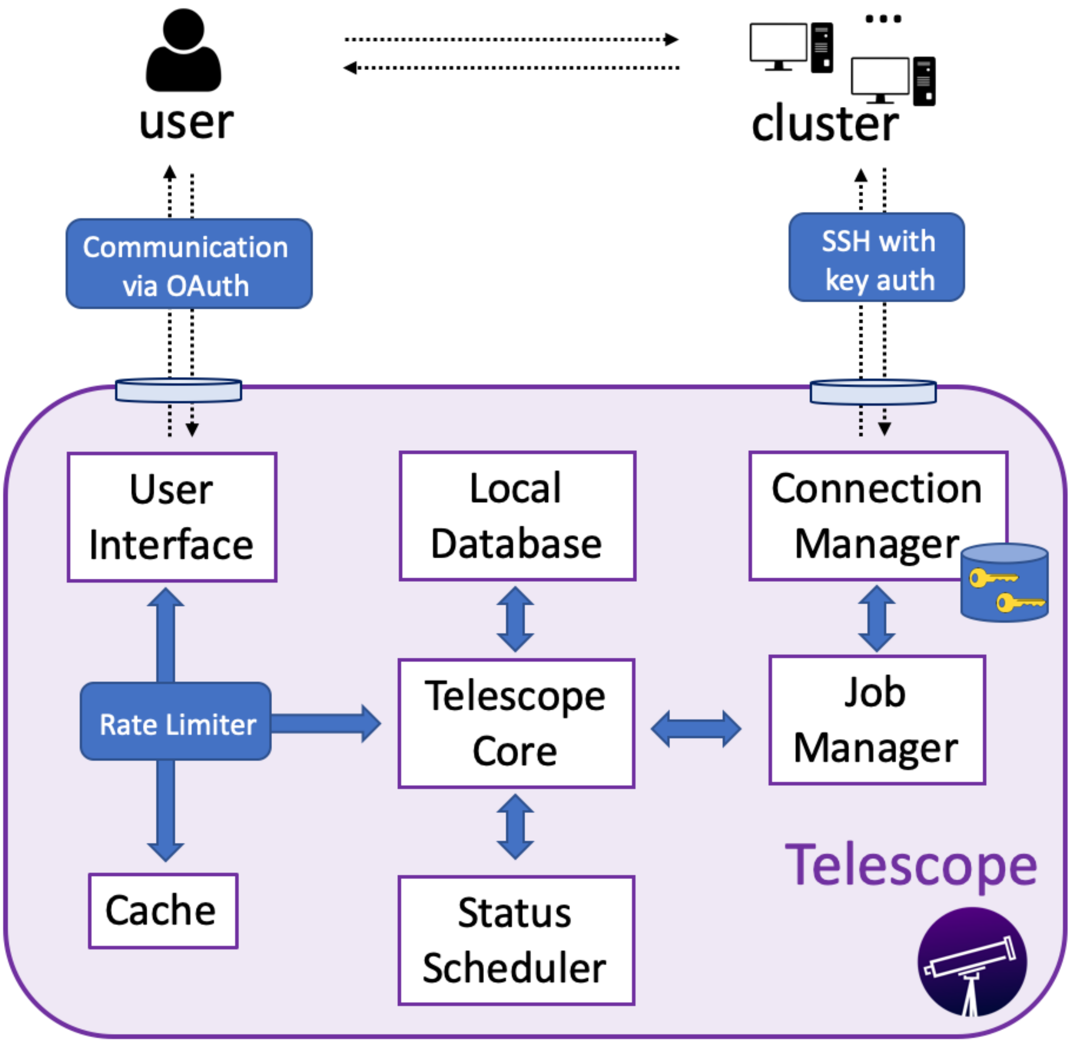
user
cluster
Communication via OAuth
SSH with key auth
User Interface
Local Database
Connection Manager
Rate Limiter
Telescope Core
Job Manager
Cache
Status Scheduler
Telescope

Figure 2.

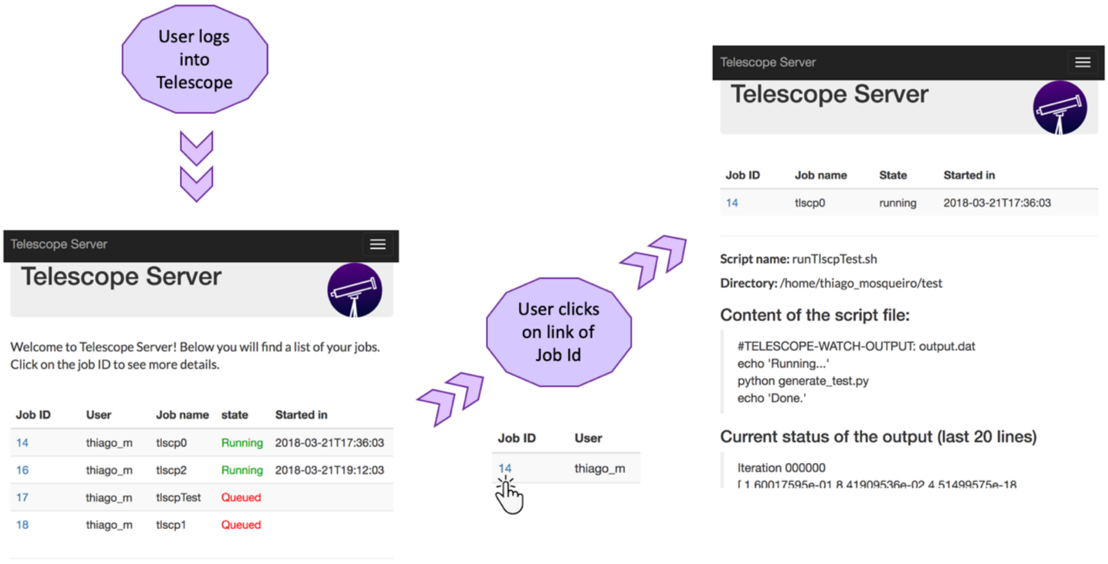
User logs into Telescope
Telescope Server
Telescope Server
Welcome to Telescope Server! Below you will find a list of your jobs. Click on the job ID to see more details.
Job ID User Job name state Started in
14 thiago_m tlscp0 Running 2018-03-21T17:36:03
16 thiago_m tlscp2 Running 2018-03-21T19:12:03
17 thiago_m tlscpTest Queued
18 thiago_m tlscp1 Queued
User clicks on link of Job Id
Job ID User
14 thiago_m
Telescope Server
Telescope Server
Job ID Job name State Started in
14 tlscp0 running 2018-03-21T17:36:03
Script name: runTlscpTest.sh
Directory: /home/thiago_mosqueiro/test
Content of the script file:
#TELESCOPE-WATCH-OUTPUT: output.dat
echo 'Running...'
python generate_test.py
echo 'Done.'
Current status of the output (last 20 lines)
Iteration 000000

Supplemental Material

Table S1. Local Database's schema, explicitly listing attributes, their corresponding data types, and descriptions. These attributes correspond to the information provided by the qstat function of scheduling system Sun Grid Engine.

**Attribute name**	**Data type**	**Description**
jobId	INTEGER (PRIMARY KEY)	Unique job id
jobName	TEXT	Name of the job
user	VARCHAR(30)	Username
status	INTEGER	Current status of job (last time qstat was updated)
path	TEXT	Path to the script that is being run
command	TEXT	Command used to submit job
sourceDirectory	TEXT	Directory from which the job was submitted
outpath	TEXT	Path and name for output file
memoryRequested	TEXT	Amount of memory requested
parallel	INTEGER	Running in parallel (1) or not (0)
cores	INTEGER	How many cores requested?
timeAdded	VARCHAR(30)	When was this entry added to the database?
runTime	TEXT	Time job has been running on cluster
timeRemaining	TEXT	Time remaining before job is killed by cluster

currentMemory	INTEGER	Memory currently in use by job
maximumMemory	INTEGER	Maximum memory used so far in job's history
clusterNode	TEXT	Node on which job was run
finalRunTime	TEXT	For finished jobs, how long did they run?
finalStatus	TEXT	How did the job end? Completed, killed, aborted?

Figure 1

Click here to download Figure Fig1-Overview.png

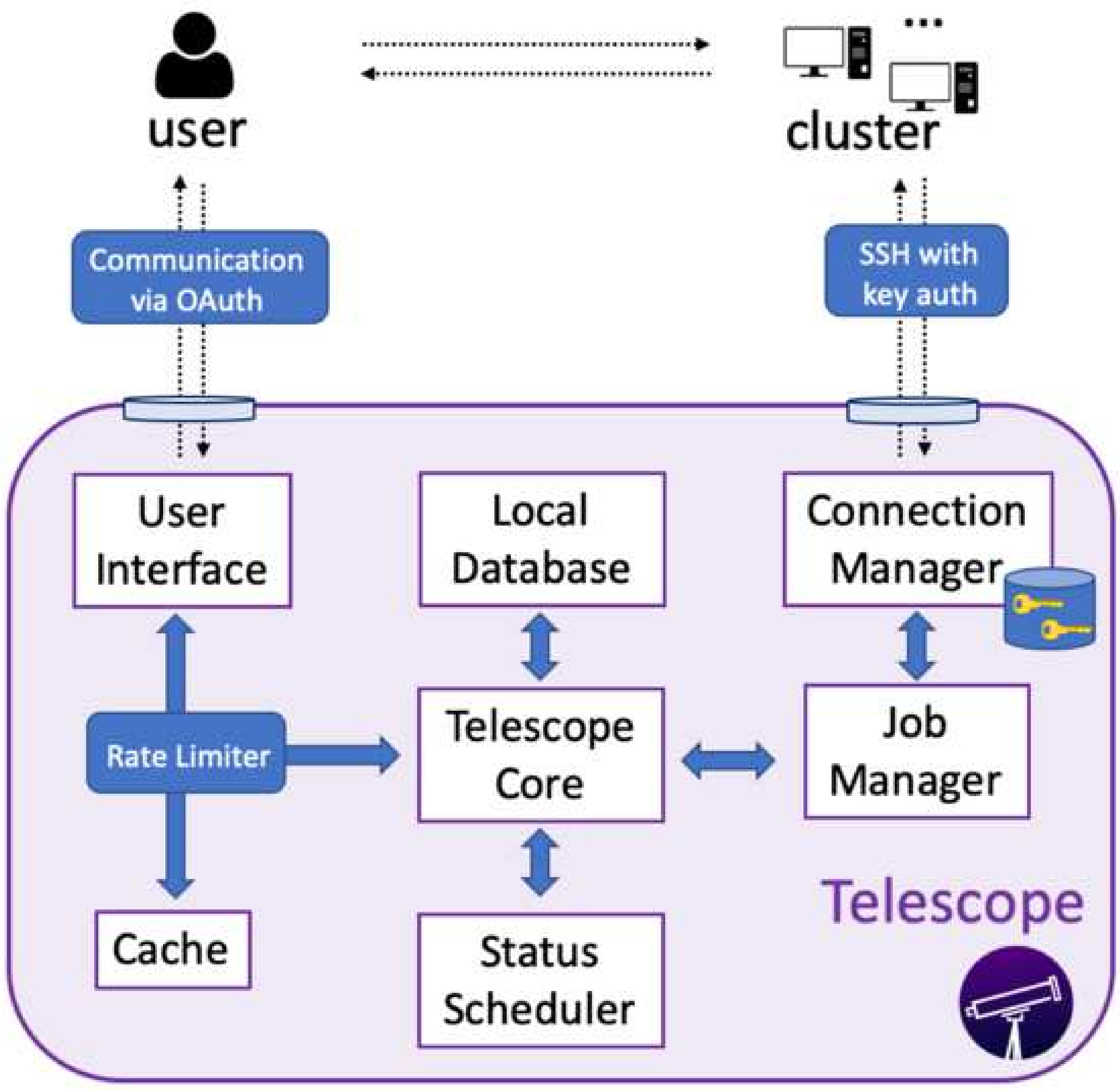

Figure 2

Click here to download Figure Fig2-Interface.png

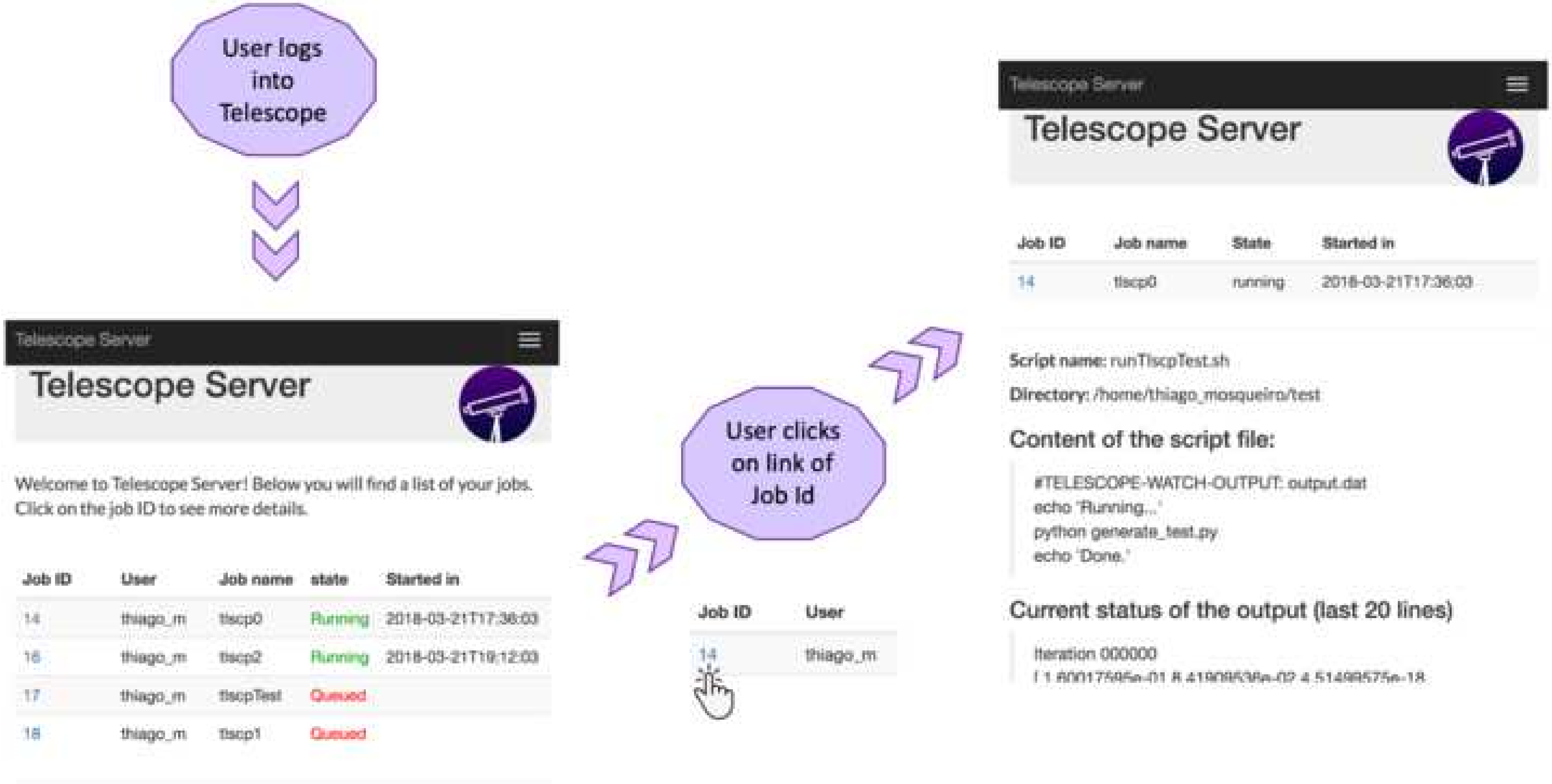

Personal Cover

Click here to download Personal Cover Telescope - Cover Letter - Giga Science.docx

USC University of Southern California

USC SCHOOL OF PHARMACY
1450 Alcazar Street
Los Angeles, CA 90089

October 2, 2019

Dear Editors of *Giga Science*,

We are delighted to submit our manuscript titled "Telescope: an interactive tool for monitoring and submitting jobs on large clusters", which we hope will be a strong candidate for consideration as a Technical Note at *Giga Science*.

Problem

The rapid advancement of genomics and sequencing technologies has generated an enormous and varied availability of new software tools and datasets in computational biology. Exponential growth in the volume of genomic sequencing data available has reshaped the landscape of contemporary biology, creating the demand for a continuous feedback loop that seamlessly integrates experimental biology and bioinformatics tools. For example, exploratory investigations may require coordinating multiple sets of experiments with the results of bioinformatics analyses using previous data resulting in a unified experimental-computational biology pipeline.

While today's high-performance computational facilities can process considerable volumes of data, they lack easy to use interfaces to supervise and adjust executed bioinformatics analysis in real-time. The current model where bioinformatics analyses are outsourced with no real-time control (for example, use of pre-cut pipelines wrapped in GUIs) is inefficient and prevent biomedical investigators from exploiting the true potential of integrating computational tools into the wet lab environment. The lack of real-time control over a user-friendly interface leaves a wide gap between bioinformatics and biological experimentation.

Solution

We have developed Telescope (https://github.com/Mangul-Lab-USC/telescope), a novel interactive protocol that interfaces with high-performance computational clusters to deliver an intuitive user interface to control and monitor in real-time bioinformatics analyses. For instance, Telescope allows users to track with their smartphones any bioinformatics tools (e.g. GATK) or jobs submitted by specific platforms (e.g. Galaxy Project), displaying in real-time partial outputs or possible warning and error messages. Telescope includes the following functionality:

1. tracking the progress and performance of running bioinformatics tools, displaying the current output in real-time in the browser;
2. interacting with the computational cluster with minimal effort, allowing cancellation and/or rescheduling of jobs with different parameters, or new jobs queuing;
3. using archived statistics about previous jobs to estimate the resources necessary for future jobs.

Telescope was designed to natively operate with a simple and straightforward interface using Web 2.0 technology compatible with most modern devices (e.g., tablets and smartphones). Moreover, Telescope assumes very little from the server-side: the existence of a scheduling system (e.g., Sun Grid Engine, SLURM, etc.) and an SSH connection, both featured in virtually all cluster systems dedicated to high-performance computing. Telescope uses PBKDF cryptography to store and employ user credentials when establishing SSH connections. To test Telescope, we deployed it as part of Hoffman2, the UCLA campus-wide computational cluster maintained by The Institute for Digital Research and Education.

Interest and significance to the broad readership of *Giga Science*

The ideas and results presented in this study help to further address the digital divide between computational and experimental efforts in contemporary biology. Our study highlights an emerging need for modern and elegant solutions to integrate the use of bioinformatics tools into the experimental environment of biomedical research. Real-time tracking allows biomedical researchers to assess partial results and identify possible problems with, for instance, the analyses of sequencing experiments. Telescope interacts with the computational resources directly, sparing the user from having to learn in-depth computer science topics and allowing biomedical researchers to leverage the power of large computational facilities in a user-friendly manner (i.e., using their own smartphones or any personal mobile device). Additionally, Telescope is domain agnostic and can be used by anyone performing extensive computational analyses (e.g,. deep learning, large-scale simulations for climate research, etc.).

Sincerely,

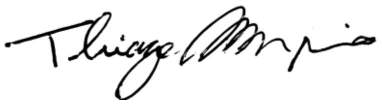

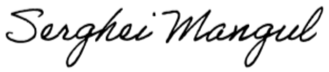

Thiago Mosqueiro
Postdoctoral researcher
University of California Los Angeles

Serghei Mangul
Assistant professor
University of Southern California
