# Supplementary material for: Telescope: an interactive tool for managing large-scale analysis from mobile devices
Source: Gigascience. 2020 Jan 23;9(1):giz163. doi: 10.1093/gigascience/giz163 (PMC6977584; doi:10.1093/gigascience/giz163)
Supplement: giz163_Supplemental_File [file giz163_supplemental_file.docx]

{Technical Note}

Telescope: an interactive tool for managing large scale analysis from mobile devices

Jaqueline J. Brito^1,†^*, Thiago Mosqueiro^2,†^, Jeremy Rotman^3^, Victor Xue^3^, Douglas J. Chapski^4^, Juan De la Hoz^5^, Paulo Matias^6^, Lana S. Martin^1^, Alex Zelikovsky^7,8^, Matteo Pellegrini^2^, Serghei Mangul^1^*

^1^ Department of Clinical Pharmacy, School of Pharmacy, University of Southern California 1985 Zonal Avenue Los Angeles, CA 90089-9121

^2^ Institute for Quantitative and Computational Biosciences, University of California Los Angeles, 611 Charles E. Young Drive East, Los Angeles, CA, 90095, USA

^3^ Department of Computer Science, University of California, Los Angeles, 404 Westwood Plaza, Los Angeles, CA 90095

^4^ Department of Anesthesiology, David Geffen School of Medicine at UCLA, 650 Charles E. Young Drive, Los Angeles, CA, 90095, USA

^5^ Center for Neurobehavioral Genetics, University of California Los Angeles, 695 Charles E Young Dr S, Los Angeles, CA, 90095, USA

^6^ Department of Computer Science, Federal University of São Carlos, km 325 Rod. Washington Luis, São Carlos, SP 13565-905, Brazil
^7^ Department of Computer Science, Georgia State University 1 Park Place, Atlanta, GA, 30303

^8^ The Laboratory of Bioinformatics, I.M. Sechenov First Moscow State Medical University, Moscow, 119991, Russia

{Supplemental Materials}

Table S1. Local Database’s schema, explicitly listing attributes, their corresponding data types, and descriptions. These attributes correspond to the information provided by the qstat function of the scheduling system Sun Grid Engine.

| Attribute name | Data type | Description |
| --- | --- | --- |
| jobId | INTEGER (PRIMARY KEY) | Unique job id |
| jobName | TEXT | Name of the job |
| user | VARCHAR(30) | Username |
| status | INTEGER | Current status of job (last time qstat was updated) |
| path | TEXT | Path to the script that is being run |
| command | TEXT | Command used to submit job |
| sourceDirectory | TEXT | Directory from which the job was submitted |
| outpath | TEXT | Path and name for output file |
| memoryRequested | TEXT | Amount of memory requested |
| parallel | INTEGER | Running in parallel (1) or not (0) |
| cores | INTEGER | How many cores requested? |
| timeAdded | VARCHAR(30) | When was this entry added to the database? |
| runTime | TEXT | Time job has been running on cluster |
| timeRemaining | TEXT | Time remaining before job is killed by cluster |
| currentMemory | INTEGER | Memory currently in use by job |
| maximumMemory | INTEGER | Maximum memory used so far in job's history |
| clusterNode | TEXT | Node on which job was run |
| finalRunTime | TEXT | For finished jobs, how long did they run? |
| finalStatus | TEXT | How did the job end? Completed, killed, aborted? |


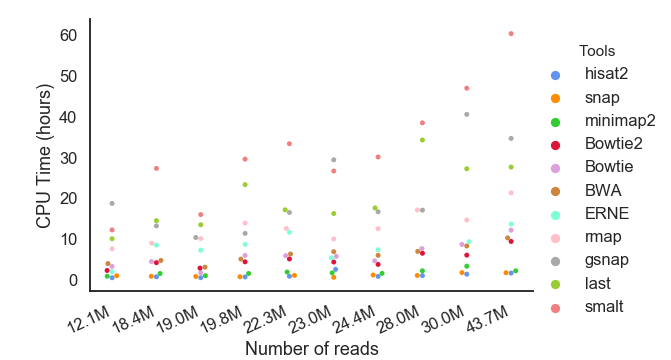


Figure S1. Comparison of the runtime (measured by CPU time in hours) for each tool against the size of each sample (measured by the number of reads).


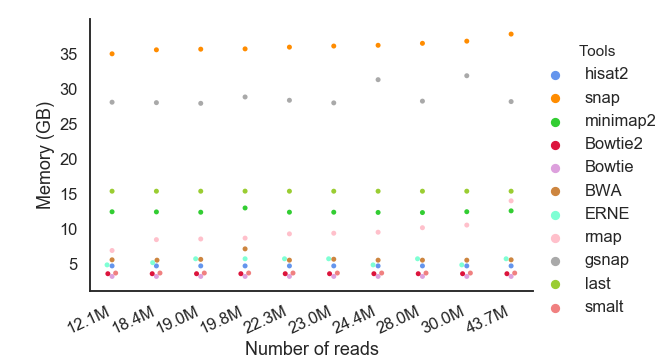


Figure S2. Comparison of the RAM (measured in gigabytes) used by each tool against the size of each sample (measured by the number of reads).

Supplemental Note 1

A number of choices can affect a job’s impact on cluster resources, including the specific bioinformatics analysis tool used and the size of the input omics dataset. To investigate the effects these specific choices have on cluster resources, we downloaded 11 read alignment tools available through Bioconda: Bowtie, Bowtie2, BWA, ERNE, gsnap, hisat2, last, minimap2, rmap, smalt, and snap. Each tool was used to align 10 whole genome sequencing (WGS) samples from the 1000 Genomes Project. All of these samples are available through the NCBI sequence read archive (SRA) with the following accessions: ERR009309 (12.1M reads), ERR013127 (23.0M reads), ERR013138 (30.0M reads), ERR045708 (43.7M reads), ERR050158 (19.8M reads), ERR162843 (28.0M reads), ERR181410 (22.3M reads), ERR183377 (18.4M reads), SRR061640 (19.0M reads), and SRR360549 (24.4M reads). For each sample and tool combination, we recorded the CPU time (Figure S1) and the RAM (Figure S2) required by the job. Number of reads was calculated by considering two Illumina sequencing paired ends as a single read.
