# Supplementary material for: Telescope: an interactive tool for managing large-scale analysis from mobile devices
Source: Gigascience. 2020 Jan 23;9(1):giz163. doi: 10.1093/gigascience/giz163 (PMC6977584; doi:10.1093/gigascience/giz163)
Supplement: giz163_Response_to_Reviewer_Comments_Original_Submission [file giz163_response_to_reviewer_comments_original_submission.pdf]

Author's Response To Reviewer Comments

Close

Please see personal cover letter for a response to each reviewer and the editor.

Close
