# Supplementary material for: Telescope: an interactive tool for managing large-scale analysis from mobile devices
Source: Gigascience. 2020 Jan 23;9(1):giz163. doi: 10.1093/gigascience/giz163 (PMC6977584; doi:10.1093/gigascience/giz163)
Supplement: giz163_Response_to_Reviewer_Comments_Revision_1 [file giz163_response_to_reviewer_comments_revision_1.pdf]

Author's Response To Reviewer Comments

Close

Dear Editors of GigaScience,

Thank you very much for conditionally accepting the second version of our paper, entitled “Telescope: an interactive tool for managing large scale analysis from mobile devices”, for publication at GigaScience. We are grateful to the reviewers for their careful assessment of the manuscript and the detailed and constructive comments. We believe that addressing these comments has produced a stronger manuscript that will hold more value for the scientific community.

Following the reviewers’ suggestions, we made one additional change to the revised version. Please find below point-by-point responses to reviewers’ comments.

Point-by-point response to reviewers’ comments

GIGA-D-19-00345R1
Telescope: an interactive tool for managing large scale analysis from mobile devices
Jaqueline J. Brito, Ph.D.; Thiago Mosqueiro, Ph.D.; Jeremy Rotman, Ph.D.; Douglas J. Chapski, Ph.D.; Juan De la Hoz, Ph.D.; Paulo Matias, Ph.D.; Lana S. Martin, Ph.D.; Alex Zelikovsky, P.hD.; Matteo Pellegrini, Ph.D.; Serghei Mangul, Ph.D.

Editor Comments: Reviewer #2 is happy with the manuscript but is wondering if you are able to clarify whether Telescope can provide more generalized help to other types of jobs? See their report below. Please also take a moment to check our website at https://www.editorialmanager.com/giga/ for any additional comments that were saved as attachments. In addition, please register any new software application in the bio.tools and SciCrunch.org databases to receive RRID (Research Resource Identification Initiative ID) and biotoolsID identifiers, and include these in your manuscript. This will facilitate tracking, reproducibility and re-use of your tool.

Author Response: Thank you for considering our revised manuscript. We address the question raised by Reviewer #2, below. We have registered our tool in both bio.tools and in SciCrunch.org, and we provide the identifiers in our manuscript. We have also ensured that our manuscript follows the formatting guidelines provided by GigaScience.

Reviewer #1: The authors responded to all my previous comments and I do not have anything else to suggest. The paper reads very well now and, in my opinion, it is ready for publication.

Author Response: Thank you for your time and prior comments.

Reviewer #2: Just a follow-up for my comment #3, authors declare that the recommendation system is an on-going project, which is totally fine. And authors show some empirical experiments with reasonable results. Well, I know making such a recommendation system or benchmarking itself can be another technical note or research product. How could the Telescope provide more generalized help to other types of jobs? For instance, does Telescope aims to provide a template, so that users themselves could choose potential software and processing types to know the resource usage? Or Telescope tries to learn historical job stats and gives rational predication. I just curious about it, please feel free not to answer if you think it is out of scope.

Author Response: We thank the reviewer for this important comment. We have added the following text to the Discussion section:

“In the future, we plan to systematically collect information about the computational resources of the

jobs run through Telescope. We will use recorded information to develop and provide a template that allows users to choose potential software and processing types to make better choices for resource usage.”

Close
