# Supplementary material for: Telescope: an interactive tool for managing large-scale analysis from mobile devices
Source: Gigascience. 2020 Jan 23;9(1):giz163. doi: 10.1093/gigascience/giz163 (PMC6977584; doi:10.1093/gigascience/giz163)
Supplement: giz163_Reviewer_1_Report_Original_Submission — Riccardo Miotto -- 10/21/2019 Reviewed [file giz163_reviewer_1_report_original_submission.pdf]

Reviewer Report

Title: Telescope: an interactive tool for managing large scale analysis from mobile devices

Version: Original Submission **Date:** 10/21/2019

Reviewer name: Riccardo Miotto

Reviewer Comments to Author:

In this technical note the authors present Telescope, a novel tool that interfaces with high-performance computational clusters to deliver an intuitive user interface for controlling and monitoring bioinformatics analyses in real-time. The paper overall is well written and the software is described properly. In my opinion, at this stage the manuscript lacks in being related with the biomedical fields. In fact, in the introduction the authors talk about applying Telescope for biomedical analyses and the text is tuned toward this use of the software. On the other hand the description of the tool seems very general and not related to the biomedical field at all (which makes sense as the tool is very general). I think the authors should try to balance better this aspect of the paper. Moreover, it is mentioned that Telescope was used at UCLA's campus-wide computational cluster but no further information is provided. It would be nice if the paper includes a description of this application, especially in terms of usage (e.g., which type of jobs where managed -- was it used for biomedical applications?).
The literature review is a little short and should probably be extended, especially strengthening more the benefits and motivations of Telescope (which are a little weak right now). I am not a big expert of this particular field but we published a paper that does something similar to run and monitor data analysis jobs for multiple myeloma patients (https://ascopubs.org/doi/full/10.1200/PO.18.00019). While our work is substantially different, it is somehow related as we also provide an interface to monitor the processes. Thus, I believe that other tools might be available in the literature that can be related to Telescope. I think the paper would be bette of some of them are mentioned, especially because this would strengthen the motivations for this work. I think mentioning some packages that enable users to create pipelines of jobs and monitoring them should be mentioned as well (e.g., Python Luigi or Airflow -- why Telescope is different?).

Level of Interest

Please indicate how interesting you found the manuscript: Choose an item.

Quality of Written English

Please indicate the quality of language in the manuscript: Choose an item.

Declaration of Competing Interests

Please complete a declaration of competing interests, considering the following questions:

- Have you in the past five years received reimbursements, fees, funding, or salary from an organisation that may in any way gain or lose financially from the publication of this manuscript, either now or in the future?
- Do you hold any stocks or shares in an organisation that may in any way gain or lose financially from the publication of this manuscript, either now or in the future?
- Do you hold or are you currently applying for any patents relating to the content of the manuscript?
- Have you received reimbursements, fees, funding, or salary from an organization that holds or has applied for patents relating to the content of the manuscript?
- Do you have any other financial competing interests?
- Do you have any non-financial competing interests in relation to this paper?

If you can answer no to all of the above, write 'I declare that I have no competing interests' below. If your reply is yes to any, please give details below.

No competing interests

I agree to the open peer review policy of the journal. I understand that my name will be included on my report to the authors and, if the manuscript is accepted for publication, my named report including any attachments I upload will be posted on the website along with the authors' responses. I agree for my report to be made available under an Open Access Creative Commons CC-BY license (http://creativecommons.org/licenses/by/4.0/). I understand that any comments which I do not wish to be included in my named report can be included as confidential comments to the editors, which will not be published.

Choose an item.

To further support our reviewers, we have joined with Publons, where you can gain additional credit to further highlight your hard work (see: https://publons.com/journal/530/gigascience). On publication of this paper, your review will be automatically added to Publons, you can then choose whether or not to claim your Publons credit. I understand this statement.

Yes Choose an item.
