# Supplementary material for: Telescope: an interactive tool for managing large-scale analysis from mobile devices
Source: Gigascience. 2020 Jan 23;9(1):giz163. doi: 10.1093/gigascience/giz163 (PMC6977584; doi:10.1093/gigascience/giz163)
Supplement: giz163_Reviewer_2_Report_Original_Submission — Shunxing Bao, Ph.D. -- 10/25/2019 Reviewed [file giz163_reviewer_2_report_original_submission.pdf]

Reviewer Report

Title: Telescope: an interactive tool for managing large scale analysis from mobile devices

Version: Original Submission **Date:** 10/25/2019

Reviewer name: Shunxing Bao, Ph.D.

Reviewer Comments to Author:

The paper introduces an interactive tool for managing large scale bioinformatics pipeline, which is publicly online. And it is tested and applied in real computation datacenter.
Please clarify the following statements:
1. The motivation of the interactional too is sun grid engine is not user-friendly. SGE is a job scheduler, and it seems that the telescope does not do deal with any configuration for configuration. For the user side, most general clients just need to create their own PBS file, and use 'qsub' to submit jobs, 'qstat' to monitor jobs and 'qdel' to delete jobs. Do you mean how to create and set up the header of the PBS file? Please clarify what the most difficulties for novice users are.
2. The authors mention that telescope can display real-time partial outputs, warnings, and error messages. In figure-2, I can see some partial sample outputs. I assume general processing outputs and error messages are from files that are defined in '#$ -o' and '#$ -e' of relevant PBS file fields. I am curious how telescope handles warning messages. Do you have any specific monitoring mechanism? Would error or warning messages trigger any notification system to tell users? If so, it should be a bonus for the telescope and should include in the manuscript.
3. The authors denote telescope can estimate resource allocation for incoming jobs. Since the code from GitHub was committed two years ago, I assume authors should have collected a bunch of job stats. I'd like to see a paragraph or figure talk about the job resource usage distribution and prove the efficiency of the telescope's estimation capability.
4. I don't quite understand the status of the scheduler's two strategies. The first one is 'returns information of all users', and the second is 'access all job data from each user.' Do you mean the second one is query by user-based? Please clarify.
5. I know this is a technical note, but could authors add some system performance results? And show some rationale for how it matters for big data analysis?

Level of Interest

Please indicate how interesting you found the manuscript: Choose an item.

Quality of Written English

Please indicate the quality of language in the manuscript: Choose an item.

Declaration of Competing Interests

Please complete a declaration of competing interests, considering the following questions:

- Have you in the past five years received reimbursements, fees, funding, or salary from an organisation that may in any way gain or lose financially from the publication of this manuscript, either now or in the future?
- Do you hold any stocks or shares in an organisation that may in any way gain or lose financially from the publication of this manuscript, either now or in the future?
- Do you hold or are you currently applying for any patents relating to the content of the manuscript?
- Have you received reimbursements, fees, funding, or salary from an organization that holds or has applied for patents relating to the content of the manuscript?
- Do you have any other financial competing interests?
- Do you have any non-financial competing interests in relation to this paper?

If you can answer no to all of the above, write 'I declare that I have no competing interests' below. If your reply is yes to any, please give details below.

I declare that I have no competing interests

I agree to the open peer review policy of the journal. I understand that my name will be included on my report to the authors and, if the manuscript is accepted for publication, my named report including any attachments I upload will be posted on the website along with the authors' responses. I agree for my report to be made available under an Open Access Creative Commons CC-BY license (http://creativecommons.org/licenses/by/4.0/). I understand that any comments which I do not wish to be included in my named report can be included as confidential comments to the editors, which will not be published.

Choose an item.

To further support our reviewers, we have joined with Publons, where you can gain additional credit to further highlight your hard work (see: https://publons.com/journal/530/gigascience). On publication of this paper, your review will be automatically added to Publons, you can then choose whether or not to claim your Publons credit. I understand this statement.

Yes Choose an item.
